# Association study between *CCR2-CCR5* genes polymorphisms and chronic Chagas heart disease in *Wichi* and in admixed populations from Argentina

**DOI:** 10.1371/journal.pntd.0007033

**Published:** 2019-01-16

**Authors:** Natalia Anahí Juiz, Elkyn Estupiñán, Daniel Hernández, Alejandra Garcilazo, Raúl Chadi, Gisela Morales Sanfurgo, Alejandro Gabriel Schijman, Silvia Andrea Longhi, Clara Isabel González

**Affiliations:** 1 INGEBI-CONICET, Buenos Aires, Argentina; 2 GIEM, Universidad Industrial de Santander, Bucaramanga, Colombia; 3 Facultad de Medicina, Universidad Nacional del Nordeste, Corrientes, Argentina; 4 Servicio de Cardiología, Hospital Pirovano, Buenos Aires, Argentina; 5 Servicio de Cardiología, Hospital Ramos Mejía, Buenos Aires, Argentina; Instituto de Ciências Biológicas, Universidade Federal de Minas Gerais, BRAZIL

## Abstract

Several studies have proposed different genetic markers of susceptibility to develop chronic Chagas cardiomyopathy (CCC). Many genes may be involved, each one making a small contribution. For this reason, an appropriate approach for this problematic is to study a large number of single nucleotide polymorphisms (SNPs) in individuals sharing a genetic background. Our aim was to analyze two *CCR2* and seven *CCR5* SNPs and their association to CCC in Argentina. A case-control study was carried out in 480 *T*. *cruzi* seropositive adults from Argentinean Gran Chaco endemic region (*Wichi* and Creole) and patients from Buenos Aires health centres. They were classified according to the Consensus on Chagas-Mazza Disease as non-demonstrated (*non-DC group*) or demonstrated (*DC group*) cardiomyopathy, i.e. asymptomatic or with CCC patients, respectively. Since, after allelic analysis, 2 out of 9 studied SNPs did not fit Hardy–Weinberg equilibrium in the unaffected *non-DC* group from *Wichi* patients, we analyzed them as a separate population. Only rs1800024T and rs41469351T in *CCR5* gene showed significant differences within *non-Wichi* population (Creole + patients from Buenos Aires centres), being the former associated to protection, and the latter to risk of CCC. No evidence of association was observed between any of the analyzed *CCR2-CCR5* gene polymorphisms and the development of CCC; however, the HHE haplotype was associated with protection in *Wichi* population. Our findings support the hypothesis that *CCR2-CCR5* genes and their haplotypes are associated with CCC; however, depending on the population studied, different associations can be found. Therefore, the evolutionary context, in which the genes or haplotypes are associated with diseases, acquires special relevance.

## Author summary

Chagas disease caused by the infection with the protozoan *Trypanosoma cruzi* is endemic in Latin America. In Argentina, it is estimated 1.5 million patients have Chagas disease and 2.2 million people in risk of *T*. *cruzi* infection. The endemic area covers the north of the country where the conditions, such as high levels of poverty and social exclusion and low population density, mostly rural, favor *T*. *cruzi* infection. Most affected people remains asymptomatic after infection for the rest of their lives, but around one third of infected people may develop clinical symptoms of visceral damage. Chronic Chagas Cardiomyopathy (CCC), the most frequent and severe consequence of the chronic infection by *T*. *cruzi*, is manifested predominately as an arrhythmogenic cardiomyopathy. The pathogenesis of CCC is not completely understood, but it is believed that the human genetic variation may be a determinant factor of disease progression. We studied in *Wichi* and in admixed populations from Argentina the *CCR2-CCR5* genes, two CC chemokine receptors involved in the trafficking of several immune cells and in the pathogenesis of cardiovascular diseases. Our results showed that *CCR2-CCR5* genes are associated with CCC and highlight the relevance of the evolutionary context in which disease-associated genes are found.

## Introduction

About one third of *Trypanosoma cruzi* chronically infected people will develop chronic Chagas cardiomyopathy (CCC). The clinical manifestations of CCC are ventricular arrhythmias, sudden cardiac death, chronic heart failure, thromboembolic phenomena and precordial chest pain [1]. The causes behind CCC development remain unclear; however, host, parasitic and environmental factors should be taken into account for a better understanding of the disease.

There is clinical manifestations heterogeneity according to the geographical region [2] and the corresponding circulating *T*. *cruzi* genotypes [3]. In this context, several association studies between parasite strain or lineages and CCC have been carried out with negative results [4, 5].Regarding host genetic characteristics, genetic markers of CCC susceptibility have been proposed [6]. In particular, research has focused on single nucleotide polymorphisms (SNPs), the most represented type of polymorphism in the human genome. As immune response and chronic inflammation are mechanisms involved in CCC, several studies have been focused on different polymorphisms in chemokines and cytokines genes as genetic markers for susceptibility to develop CCC. They have been carried out in endemic countries such as Peru [7], Mexico [8, 9], Colombia [10–15], Brazil [16–19] and Bolivia [20]. In Argentina, where approximately one million and a half people are infected (4% of the total population), with a prevalence in endemic areas greater than 60% [21], until now only there were association studies for HLA class II *DRB1* alleles with CCC [22, 23], but not for those immunological genes mentioned above.

Previously case-control studies conducted in Santander, Colombia, have identified SNPs significantly associated with CCC severity in *CCR2* and *CCR5 loci* [15, 24], which encode two CC chemokine receptors involved in the trafficking of leukocytes and in cardiovascular diseases pathogenesis [25]. It is essential to make replication studies of genotype–phenotype associations for establishing their direct relationship with the disease [26].

Thus, the aim of this study was to analyse SNPs in *CCR2* and *CCR5* and their association with CCC in patients who attend to health centers of Buenos Aires and populations from endemic area as the Gran Chaco ecoregion, with a high prevalence of infection.

## Methods

### Ethics statement

The research protocols followed the tenets of the Declaration of Helsinki and Guidelines according to Resolution N°1480/11 of the “Ministerio de Salud” from Argentina and were approved by the Local Medical Ethics Committees named Committee of The Institute of Regional Medicine of the Northeastern National University (UNNE), Resistencia, Chaco; IDACH (Chaco Aboriginal Institute); Committees of Ramos Mejía and Pirovano Hospitals from Buenos Aires, upon written informed consents of adult individuals.

### Study populations

A case-control study has been carried out including 480 individuals serologically positive for *T*. *cruzi* antigens inhabiting in endemic zone of the Provinces of Chaco and Formosa, i.e. Argentinean Gran Chaco Region, and in patients of health centers of Buenos Aires from April 2012 to November 2017. They were classified according to the Consensus on Chagas-Mazza Disease [27] as individuals with non-demonstrated cardiomyopathy (*non-DC* group) or with demonstrated cardiomyopathy (*DC*-group). The *non*-DC group, which represents the control group, were individuals with chronic infection but lacking clinical symptoms: they do not show obvious pathological signs during the cardiovascular physical examination and the complementary studies performed (electrocardiogram, stress test, etc.) were normal as established for each practice. The *DC*-group, which represents the case group, was constituted by seropositive individuals with clinical symptoms and electrocardiography alterations.

### Genotyping

Genomic DNA was isolated from 400 μL of EDTA anticoagulated peripheral blood sample using the DNA extraction kit (High Pure PCR Template Preparation Kit, Roche) and SNPs were determined by TaqMan 5´ allelic discrimination assay method performed by Applied Biosystems. The SNPs studied were: rs1799864 and rs3138042 in *CCR2* and rs2856758, rs2734648, rs1799987, rs1799988, rs41469351, rs1800023 and rs1800024 in *CCR5*.

### Statistical analysis

The control group (*non-DC* group) was tested for all markers on Hardy–Weinberg equilibrium (HWE) and a p-value <0.01 was considered as evidence of deviation from HWE. HWE, frequencies, odds ratios (OR), their 95% confidence intervals (CIs) and the genetic effect of each polymorphism in CCC, assessed by logistic regression model with cases or controls (*DC* and *non-DC* groups, respectively) as the dependent variables were calculated using PLINK V1.07 software [28]. As age and gender are possible confounding variables they were included as additional covariates in the analysis and p-values were adjusted for multiple testing by Bonferroni. Wright’s fixation coefficient to establish population’s structuring were determined by the Arlequin 3.11 software [29]. Correspondence analyses were carried out using R statistical package and pairwise linkage disequilibrium (LD), coefficient of linkage disequilibrium (D´) and haplotypes were estimated with an expectation–maximization algorithm implemented the Haploview 4.2 software [30]. The statistical power of our study was calculated with the Genetic Power Calculator for one-stage case–control studies. (http://zzz.bwh.harvard.edu/gpc/) [31].

## Results

### Population characterization

The samples included patients from Buenos Aires health centres and individuals from rural endemic region localities: Río Muerto, Las Hacheras, Misión Nueva Pompeya, Miraflores (Chaco) and Las Lomitas, Pozo del Tigre, Laguna Yema, Estanislao del Campo (Formosa). After genotype frequencies analysis in the control group (*non-DC*), two out of 9 genotyped *loci*, rs1799864 and rs1800024, did not fit HWE (p<0.002 and 0.005, respectively) and the Fst fixation coefficient showed moderate genetic differentiation of the population (Fst = 0.102). As in the endemic surveyed region co-habit different communities and two of them (Native American *Wichis* and Creoles) were included in this study, we decided to divide our population in three potential subpopulations: patients from Buenos Aires (n = 202), Native American *Wichis* (n = 144) and Creoles (n = 134).

**Fig 1:**
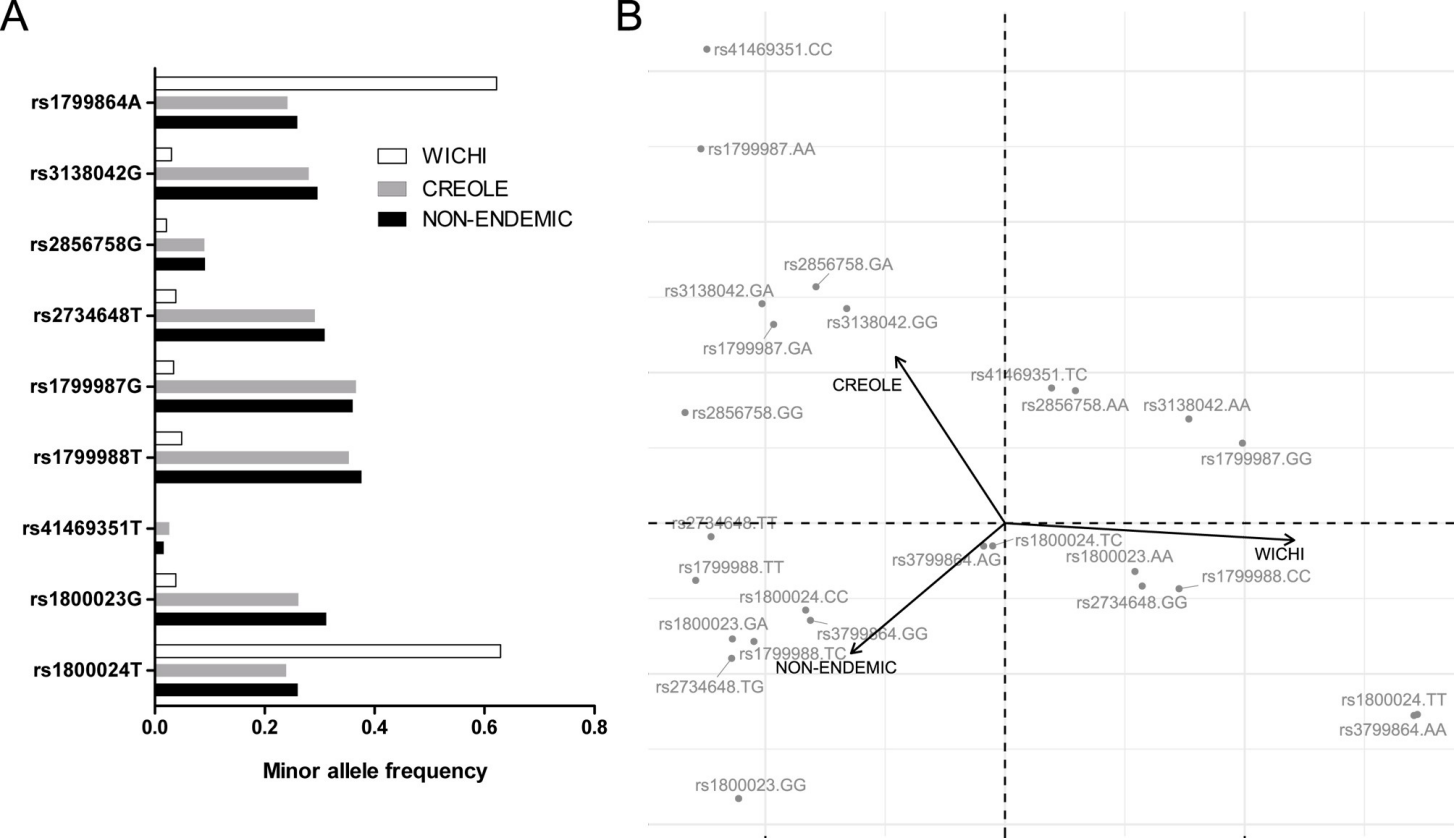
**(A) Minor allele frequencies for SNPs in *CCR2-CCR5* gene region in three subpopulations from Argentina**: Patients from Buenos Aires centres (non-endemic), and two populations from endemic region, Creole and *Wichi*. (**B) Correspondence analysis.** Genotypes for each polymorphic site and populations are represented in a two dimensional plot.

We performed allele frequencies comparison, considering both *DC* and *non-DC* groups, among the three subpopulations (Creole, *Wichi* and patients from Buenos Aires centres) by Chi-square test of homogeneity. This test demonstrated that allele frequencies were the same for Creole and patients from Buenos Aires centres, but they all differ from *Wichi* subpopulation (p-values<0.05). Fig 1A shows the minor allele frequency (MAF) for the three potential subpopulations. Moreover, the correspondence analysis also indicated population structure (Fig 1B), as the first dimension explained 83.39% of the variance. In concordance with the previous results this analysis showed that genotype frequencies of the Gran Chaco *Wichi* population differ to the Creole population from the same endemic region and also to the Buenos Aires patients (non-endemic). However, these last two groups (Creoles and patients that lived in Buenos Aires), which share a Caucasian genetic background and have similar genotype frequencies, were considered as a same population that was named as “*non-Wichi* population” (n = 336). Thus, the subsequent association analyses between *non-DC* and *DC groups* were carried out independently in these two subpopulations: *non*-*Wichi* and *Wichi*.

Ages in *non-DC* and *DC* groups within *non-Wichi* or *Wichi* populations were similar (p = 0.0642 and 0.249, respectively), while significant differences were observed between populations (p <0.0001) (Table 1). Regarding to gender, populations were homogeneous samples (p = 0.08 and 0.2837, *non-Wichi* or *Wichi* samples, respectively) (Table 1).

**Table 1:** Characterization of the populations studied.

Variables		*non-Wichi*		*Wichi*	
		*DC*	*non-DC*	*DC*	*non-DC*
**Age (years)**	**Median [rank]**	56 [17–85]	52.5 [17–81]	36 [16–82]	32 [17–81]
	**Mean ± SD**	54.14 ± 12.76	51.30 ± 15.15	38.40 ± 14.72	35.43 ± 14.04
**Gender (n)**	**Male (n/%)**	93/54.7	75/45.2	26/57.7	47/47.5
	**Female (n/%)**	77/45.3	91/54.8	19/42.2	52/52.5
	**Total (n)**	170	166	45	99

Regarding the clinical characteristics of these populations, *Wichi* patients presented normal vital signs, regular pulse, although episodes of dizziness and normal systolic blood pressure or hypotension were observed. In general, they were asymptomatic patients, and only the presence of palpitations or dyspnea were found in aborigines over 60 years of age. The EKG showed a heart rate of less than 60 beats per minute; therefore sinus bradycardia and left anterior hemiblock could be detected. Instead, *non-Wichi* patients presented paroxysmal palpitations, dyspnea on exertion, paroxysmal nocturnal dyspnea and syncope. A higher prevalence of chronic decompensated heart failure and dilated cardiomyopathy as well as hypertensive patients were observed, with systolic blood pressure greater than 140 mmHg and diastolic blood pressure greater than 90 mmHg. In the EKG, these patients presented ventricular extrasystoles, with a greater presence of complete right bundle branch block than left anterior hemiblock and also bifascicular block with ventricular premature beats. These intraventricular disorders appeared at younger ages compared to aboriginal patients.

### Allelic and genotypic association analysis

In *non-Wichi* population the only SNPs with significant differences (Table 2) in the allelic analysis were: rs1800024T, p = 0.041; OR = 0.69 (0.49–0.99), with a lower frequency of 0.218 in *DC* compared to 0.286 in *non-DC* group; and rs41469351T with frequencies of 0.038 and 0.008 in *DC* and *non-DC* groups, respectively (p = 0.028; OR = 4.88 (1.03–23.24)) (Table 2). No differences were observed in genotype frequencies between groups.

**Table 2:** Relative genotype and allele frequencies for *non-Wichi* and *Wichi* populations.

SNP			*non-Wichi*			*Wichi*		
			*DC* n = 170	*non-DC* n = 166	p_adjusted_	*DC* n = 45	*non-DC* n = 99	p_adjusted_
***CCR2***	**rs1799864**	**AA**	0.082	0.062	0.359	0.534	0.394	0.241
		**AG**	0.324	0.395		0.332	0.384	
		**GG**	0.594	0.543		0.134	0.222	
		**A**	0.244	0.259	0.653	0.7	0.586	0.064
		**G**	0.756	0.741		0.3	0.414	
	**rs3138042**	**GG**	0.077	0.073	0.994	-	-	NA
		**GA**	0.423	0.423		0.1	0.038	
		**AA**	0.5	0.504		0.9	0.962	
		**G**	0.288	0.285	0.937	0.05	0.02	0.187
		**A**	0.712	0.715		0.95	0.98	
***CCR5***	**rs2856758**	**GG**	0.01	0.008	NA	-	-	NA
		**GA**	0.144	0.179		-	0.064	
		**AA**	0.846	0.813		1	0.936	
		**G**	0.082	0.098	0.568	-	0.032	0.106
		**A**	0.918	0.902		1	0.968	
	**rs2734648**	**TT**	0.124	0.054	0.083	0.022	-	NA
		**TG**	0.412	0.44		0.089	0.051	
		**GG**	0.465	0.506		0.888	0.949	
		**T**	0.329	0.274	0.128	0.067	0.025	0.089
		**G**	0.671	0.726		0.933	0.975	
	**rs1799987**	**GG**	0.173	0.106	0.319	-	-	NA
		**GA**	0.423	0.48		0.074	0.064	
		**AA**	0.404	0.415		0.926	0.936	
		**G**	0.385	0.346	0.398	0.038	0.032	0.827
		**A**	0.615	0.654		0.962	0.968	
	**rs1799988**	**TT**	0.182	0.103	0.096	0.022	-	NA
		**TC**	0.412	0.485		0.067	0.092	
		**CC**	0.406	0.412		0.911	0.908	
		**T**	0.388	0.345	0.251	0.054	0.045	0.712
		**C**	0.612	0.655		0.946	0.955	
	**rs41469351**	**TT**	-	-	NA	-	-	NA
		**TC**	0.077	0.016		-	-	
		**CC**	0.923	0.984		1	1	
		**T**	0.038	0.008	0.028*	-	-	NA
		**C**	0.962	0.992		1	1	
	**rs1800023**	**GG**	0.106	0.048	0.141	0.022	-	NA
		**GA**	0.418	0.44		0.089	0.051	
		**AA**	0.476	0.512		0.888	0.949	
		**G**	0.315	0.268	0.184	0.067	0.025	0.089
		**A**	0.685	0.732		0.933	0.975	
	**rs1800024**	**TT**	0.065	0.078	0.071	0.511	0.414	0.258
		**TC**	0.306	0.416		0.377	0.363	
		**CC**	0.629	0.506		0.112	0.222	
		**T**	0.218	0.286	0.041*	0.7	0.596	0.09
		**C**	0.782	0.714		0.3	0.404	

NA: not applicable

*p < 0.05

No association was found in none of the studied SNPs in allele frequencies within the *Wichi* population (Table 2). The association test could only be implemented in rs1799864 and rs1800024.

### Haplotype analysis

**Fig 2:**
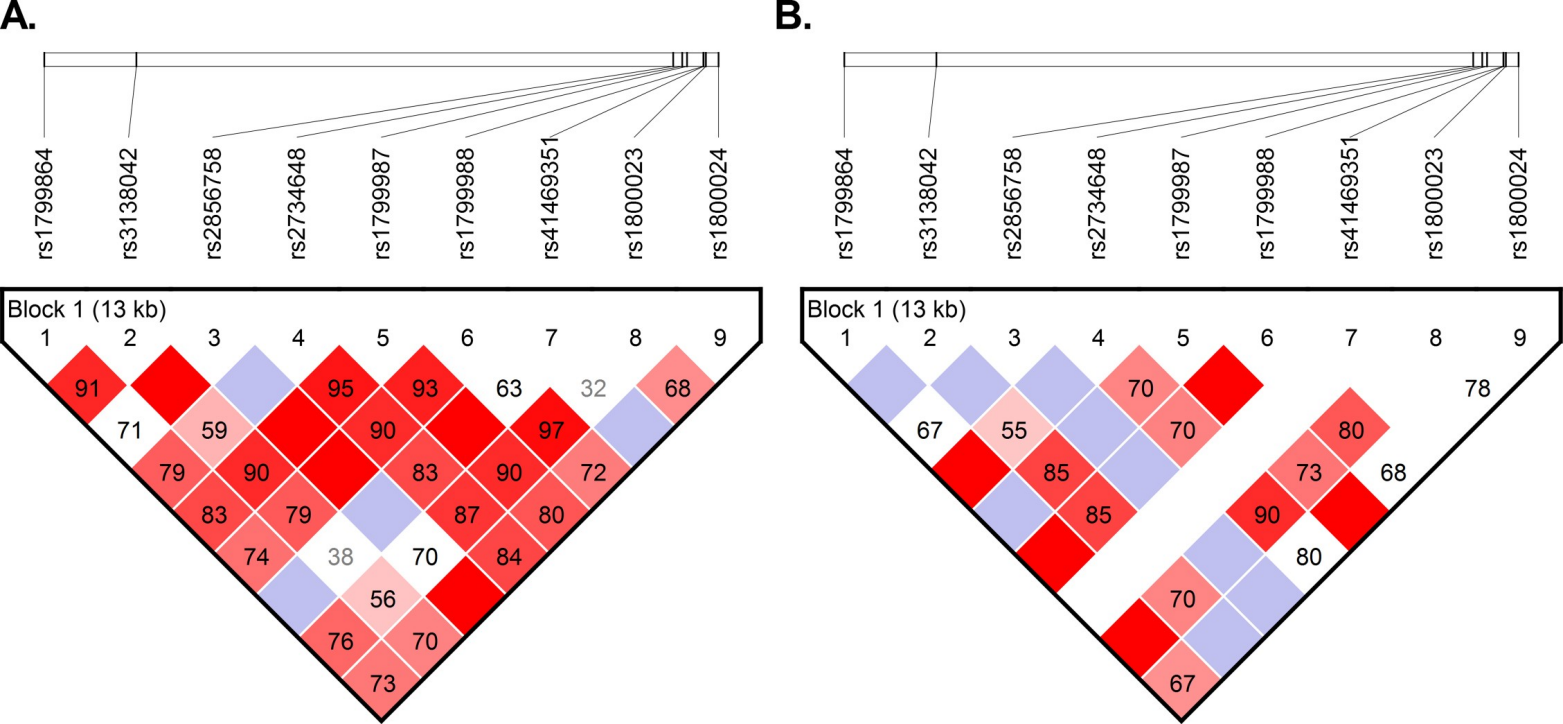
LD plot across *CCR2*-*CCR5* region. A high-resolution LD among SNPs studied in *non-Wichi* (**A**) and *Wichi* (**B**) populations. D´ values are reported in the boxes and represented such a colour scale from red (higher D´scores) to white colour (lower D´ scores).

To evaluate linkage disequilibrium between pairs of SNPs in *CCR2*-*CCR5* region, an analysis using Haploview was performed. Both populations showed linkage disequilibrium among studied SNPs (Fig 2).

Haplotypes were constructed based on the evolution of linked *CCR2* and *CCR5* mutations, including only the rs1799864 for *CCR2* and the seven *CCR5* SNPs, as it was previously defined for HIV-1 studies association [32]. As the SNP rs3138042 does not take part of the haplotypes already described by Mummidi *et al*. [32], it was excluded for this analysis.

HHE and HHC were the most represented haplotypes in *non-Wichi* population and none described haplotypes showed significant differences between *DC* and *non-DC* groups (Table 3). Among *Wichi*, HHF*2 was the most frequent haplotype followed by HHE, with a higher frequency in *non-DC* than *DC* group (p = 0.022; OR = 0.49 (0.29–1.23)) (Table 3).

**Table 3:** Haplogroup frequencies.

Haplotype	^a^1	3	4	5	6	7	8	9	*non-Wichi Frequencies*			*Wichi Frequencies*		
									Total	*DC*. *non-DC*	p	Total	*DC*. *non-DC*	p
**HHA**	G	A	G	G	T	C	A	C	0.056	0.049. 0.063	0.41	0.014	0.011. 0.016	0.768
**HHB**	G	A	T	G	T	C	A	C	0.004	0.001. 0.008	0.16	0	0	-
**HHC**	G	A	T	G	T	C	G	C	0.24	0.260. 0.219	0.214	0.024	0.044. 0.015	0.144
**HHD**	G	A	T	G	T	T	A	C	0.014	0.015. 0.013	0.819	0	0	-
**HHE**	G	A	G	A	C	C	A	C	0.278	0.278. 0.279	0.965	0.236	0.151. 0.275	0.022*
HHF*1	G	A	G	A	C	C	A	T	0.031	0.028. 0.034	0.674	0.076	0.082. 0.073	0.786
HHF*2	A	A	G	A	C	C	A	T	0.189	0.167. 0.213	0.131	0.543	0.595. 0.520	0.236
**HHG**	G	G	G	A	C	C	A	C	0.077	0.067. 0.086	0.365	0.015	0.000. 0.022	0.152

a) 1 = rs1799864; 3 = rs2856758; 4 = rs2734648; 5 = rs1799987; 6 = rs1799988; 7 = rs41469351; 8 = rs1800023; 9 = rs1800024

Grey boxes denote a base change in the haplogroup respect to the ancestral HHA haplotype.

*p < 0.05

## Discussion

Population genetic structure is the consequence of previous demographic events and gene and culture co-evolution [33–35], both with different rates and rules. A study in human mummies showed that *T*. *cruzi* transmission among infected wild reservoirs was probably established in the first moment of Andean coast human colonization [36], suggesting that *T*. *cruzi* and humans co-evolved from over 9000 years and thus, south Amerindians may have developed a peculiar way of dealing with *T*. *cruzi* infection [37]. Thus, host-pathogen interaction is another factor to be taken into account for understanding population genetic evolution.

*Wichi* is one of the Argentinian native populations, with its own cultural patterns, that inhabit the Impenetrable Chaqueño, a region with extreme climatic conditions and scarce urban centres communication. This isolation results in a genetic differentiation from other populations [37, 38]. In concordance with this data, our results in some *T*. *cruzi* seropositive Argentinian populations showed that the *Wichi* community exhibits different allele frequencies compared to the Impenetrable Chaqueño Creole population which is genetically similar to patients from Buenos Aires centres (admixed populations) in the studied *locus*. Moreover, the correspondence analysis indicated a clear genotype frequencies differentiation between *Wichi* and the other sub-populations: in *Wichi* 4 out of 9 SNPs showed allele frequencies close to zero and for the two SNPs located at both extremes of the gene region studied (rs1799864 and rs1800024) the most represented alleles were not the ancestral. These results reinforced the subdivision in *Wichi* and *non-Wichi* groups for genetic analysis and highlight the importance of studying the different genetic backgrounds of Amerindian populations. Despite limitations related to both *Wichi* and *non-Wichi* sample sizes and since the prevalence of Chagas disease in endemic areas is greater than 60% [39], statistical power calculation of our study showed that subdivision in two groups have a power of 80% (S1 Table).

Chemokines have been associated not only with the initial control of *T*. *cruzi* infection, but also the maintenance of chronic inflammation resulting from the inability of immune system to eliminate the parasite [40]. In different studies, common *CCR5* SNPs were analysed individually with variable results. In Colombian population was found that rs2856758G, within the *CCR5* promoter region, was associated with a reduced risk of susceptibility to develop CCC [24]. In our populations this allele has a very low frequency compared to that observed among Colombians, suggesting a possible reason why the association is not replicated. In this work, like in Flórez *et al*. [24], we found no evidence of association of both rs1799987 and rs1799988 variants which had been previously described in Peruvian and Venezuelan [41, 42]. This discrepancy might be the result of lower size sample in the last reports (less than a hundred patients), with a higher probability of positive false.

Our results also showed a significant rs1800024T decreased frequency in *non-Wichi* population *DC* group suggesting protection to CCC, while Machuca *et al*. [15] observed in Colombians that it correlated with CCC severity. Rs1800024T is the allele that characterizes the HHF haplotype (HHF*1 and HHF*2) which is associated with higher levels of CCR5 expression [32] as a consequence of a differential binding of T and G alleles to certain nuclear factors, especially factors of the NFkB family. Likewise, *in-silico* studies predict that the T allele, together with other transcription factors such as IRF1 receptors, are involved in the expression of innate immunity proteins which are important in parasite control and therefore less inflammation related [32]. Taken together, these results could indicate that higher levels of CCR5 expression might protect from cardiomyopathy development by decreasing the parasitic load or allowing parasite control. Meanwhile in those individuals for whom this protection is insufficient, the parasite and antigenic persistence would stimulate a chronic inflammation responsible for more severe tissue damage. Something similar could happen in the case of rs2734648T which binds to nuclear factors with more avidity than the G allele [32] and this might also alter CCR5 protein expression levels. Here we found in the case of *non-Wichi* population an association between this SNP and CCC in subjects with 2 copies of this allele (rs2734648TT genotype, recessive model, p = 0.026) while in Colombians it was associated with less severity [24].

Regarding the rs41469351T, its frequency was very low both in Colombian and Argentinian *non-Wichi* populations, and null among *Wichis*. In the *non-Wichi* population we found a higher frequency of this allele in the *DC* group, although this significance might be taken carefully because of the low frequency values.

Previous studies have shown that several polymorphic changes in the *CCR5* promoter, that define the haplotypes, may influence differently both expression levels of *CCR5* surface and proportions of peripheral blood cells expressing this protein [32]. In our study, the HHE haplotype was the most common haplotype in *non-Wichi* population followed by HHC and HHF*2. These three haplogroups were also the most frequent in Colombian population [15, 24], although none of them were associated with CCC in *non-Wichi* group. In contrast, in *Wichi* individuals only the frequencies of two haplotypes were higher than 0.1, being HHF*2 haplotype the highest frequent followed by HHE, the latter associated with protection from CCC. Interestingly, the HHC frequency -one of the most frequent haplotypes in other populations- is considerably low among *Wichis*. This haplogroup together with HHA have been associated with a decreased promoter activity, while HHE and HHF are characterized by a higher activity. Therefore, a higher expression level of *CCR5* and *CCR2* could favour a greater control of infection with *T*. *cruzi* and thus would be less susceptible to develop CCC. These findings, together with the absence of rs41469351T, could be some factors involved in a lower prevalence of right bundle branch block, one of the anomalies mostly observed in the electrocardiograms of CCC patients, reported in *Wichi* ethnicity compared to Creole (1.8% vs 10%, respectively, OR = 6.0) [39].

It is important to note that both in the *Wichi* and in the *non-Wichi* populations it is possible to find individuals who manifest the disease at very young ages (Table 1). That is, the clinical onset seems to be independent of the genetic background, but risk of susceptibility to developing CCC would not be. Although our study presents some limitations in sample size our results are valuable as they described a unique population with a large history of co-evolution with the parasite.

In summary, our findings support the hypothesis that *CCR2-CCR5* genes and their haplotypes are associated with CCC and this study also highlight the importance of considering the evolutionary context in which disease-associated genes or haplotypes are found and to underline the possible impact of allele–allele interactions, especially among alleles with different evolutionary histories.

## Supporting information

S1 Table Statistical power calculation of our study considering three different OR. (DOCX)

S2 Table STROBE checklist case-control. (DOC)
