# Supplementary material for: Association study between CCR2-CCR5 genes polymorphisms and chronic Chagas heart disease in Wichi and in admixed populations from Argentina
Source: PLoS Negl Trop Dis. 2019 Jan 16;13(1):e0007033. doi: 10.1371/journal.pntd.0007033 (PMC6334923; doi:10.1371/journal.pntd.0007033)
Supplement: S1 Table — (DOCX) [file pntd.0007033.s001.docx]

**S1 Table**

**Statistical power calculation of our study considering three different OR**

|  | N cases for 80% power | |
| --- | --- | --- |
|  |  |  |
|  | *non-Wichi* | *Wichi* |
|  | *non-DC / DC* | *non-DC / DC* |
|  | (166/170) | (99/45) |
| OR=1.5 | 61 | 24 |
| OR=1.25 | 208 | 78 |
| OR=1.1 | 1171 | 470 |
|  |  |  |

The estimation was performed considering a minor allele frequency of 25% and a prevalence of 62% and 71% for non-*Wichi* and *Wichi*, respectively.
